# Identifying maternal needs following childbirth: comparison between pregnant women and recent mothers

**DOI:** 10.1186/s12884-021-03858-7

**Published:** 2021-05-28

**Authors:** Justine Slomian, Jean-Yves Reginster, Patrick Emonts, Olivier Bruyère

**Affiliations:** 1 Department of Public Health, Epidemiology and Health Economics, University of Liège, Quartier Hôpital, Avenue Hippocrate 13, Bât. B23, 4000 Liège, Belgium; 2 Division and Head of the Public Health Department, Bone and Cartilage metabolism Department, CHU Liège, Quai Godefroid Kurth 45, 4000 Liège, Belgium; 3 Division of Public Health, Epidemiology and Health Economics, University of Liège, Avenue Hippocrate 13, Bât. B23, 4000 Liège, Belgium

**Keywords:** Maternal needs, Postpartum period, Information, Practical support, Psychological support, Sharing experience

## Abstract

**Background:**

The postnatal period is associated with new needs for mothers. Four categories of needs were highlighted in a previous study: for information, for psychological support, for the sharing of experiences and for practical and material support. To ensure that these four needs are inherent to the postpartum period, the aims of this study is to examine these needs by comparing recent mothers’ needs with the needs of pregnant women.

**Methods:**

The 4 needs previously identified were cross-sectionally investigated by online self-reported questionnaires completed by women in their last trimester of pregnancy and by mothers who had a child between 0 and 6 months of age.

**Results:**

The 4 needs were largely present during the postpartum period. The need for information seemed to be more present during pregnancy (92.4 %) than during the postpartum period (84.6 %, *p *= 0.03), but women used the Internet significantly more often to search for information after childbirth (54.8 %) than during pregnancy (41.2 %, *p* < 0.0001). The needs for psychological support and to share experiences seemed to be closely linked. Even if the global satisfaction with psychological support was fairly high, it weakened after childbirth (*p* < 0.05). Feelings of loneliness (*p* < 0.0001) and depression scores (*p *= 0.01) were also higher during the postpartum period than during pregnancy. Finally, the need for practical support was also more pronounced during the postpartum period than during pregnancy (*p* = 0.01).

**Conclusions:**

All mothers seem to meet the 4 identified needs during the postpartum period but at different levels of intensity. Trying to meet these needs could offer an opportunity to improve mothers’ quality of life.

## Background

The transition to motherhood is one of the greatest changes in a woman’s life [1, 2]. The postnatal period is a stressful time that brings not only physical but also psychological and social changes [3, 4]. After childbirth, the “maternal psyche” invades the entire psychic life of women, determining their thoughts and behaviors and leading them to take care of their babies instead of taking care of themselves [5, 6]. Mothers must therefore address these sudden and intense changes in their roles and responsibilities [7–9].

Thus, the postnatal period is associated with new needs for mothers. A previous study in our department evaluated the needs of mothers in the year after childbirth from a qualitative perspective [10]. Four categories of needs were highlighted: needs for information, for psychological support, for the sharing of experiences and for practical and material support. Primiparous and multiparous women seemed to have similar needs but at different levels of intensity. For example, this previous study showed that primiparous women perceived a greater lack of information from many perspectives (medically, administratively, regarding existing services, feeling unprepared for motherhood, reliability of information, etc.) than did multiparous women [10]. Indeed, the need for information appeared to decrease and become more specific for subsequent children. In attempting to meet this need, mothers are increasingly turning to the Internet to help themselves make decisions and manage their postpartum lives [10–16]. With respect to the needs for psychological support and the sharing of experience, it has already been shown that women often have many fears and anxieties regarding early motherhood and their changing role [10, 17] and perceive a lack of control over their lives, incomplete maternal feelings and unstable relationships with their husbands and others [4, 10]. Women therefore need to be surrounded, reassured and understood by those who will emotionally support them, especially by the fathers of their children but also by other mothers [10, 18–20]. In addition, isolation and feelings of loneliness recur during the postnatal period [10]. Finally, the majority of women expressed the need for help with housework chores, especially in the first few postpartum weeks [10].

This previous study used a qualitative method that was well adapted to explore the mothers’ needs during the postnatal period and to obtain very rich results allowing participants to express themselves freely and spontaneously [21]. Nevertheless, one limitation of qualitative investigations is that, even if they focus on the particularities and allow for the extraction of tendencies from the collected interviews, they do not allow for the measurement of the frequencies of the themes highlighted. Thus, it was not possible to generalize the results. For this reason, it was also important to explore these needs quantitatively on a larger scale. To ensure that these four needs are inherent to the postpartum period, it is important to explore and compare these needs between pregnant women and women who have given birth. Therefore, the aim of the present study was to examine the 4 previously identified postnatal needs (for information, for psychological support, for the sharing of experiences and for practical and material support [10]) by comparing the mothers’ needs with the needs of pregnant women. In addition, to our knowledge, there is no study that compares maternal needs during and after pregnancy.

## Methods

### Study design

The 4 needs previously identified (for information, for psychological support, for the sharing of experiences and for practical and material support [10]) were cross-sectionally investigated by online self-reported questionnaires among women in their last trimester of pregnancy and among mothers who had a child between 0 and 6 months of age. This study received ethics approval from the “Comité d’Ethique Hospitalo-Facultaire Universitaire de Liège” under the number 2017/14. A complete written description of the research, including intends to publish, was provided to the participant at the beginning of the first questionnaire. This information page had to be approved to continue to the questionnaire. Participation in the study was therefore considered to indicate the consent of the respondent. Participation in the survey was therefore confidential.

### Participants and sample

All the pregnant women with at least 32 weeks of amenorrhea and all the mothers who had a child between 0 and 6 months of age were included in this study. The inclusion criteria were as follows: being pregnant for at least 32 weeks or having a child between 0 and 6 months of age; giving birth in Belgium; understanding French; having any access to the Internet (at home, at work, via a smartphone, etc.); and agreeing to participate in the study. The exclusion criteria were foetal death in utero, very premature childbirth (< 34 weeks of gestation), foetal pathologies and multiple pregnancies.

### Questionnaires and parameters investigated

A literature review highlighted that none of the published instruments were able to meet the objectives of this research. A questionnaire was therefore designed specifically for this study. Indeed, the objective of the present study was to examine the mothers’ needs in regard to the result of the previous study conducted by our department, namely, the needs for information, for psychological support, for the sharing of experiences and for practical and material support [10]. There were two types of questionnaires: one questionnaire for pregnant women from 32 weeks of gestation until birth and one questionnaire for mothers who had children between 0 and 6 months of age. These two questionnaires contained similar questions to evaluate the mothers’ needs and specific questions about pregnancy or birth with respect to the different cases.

Questionnaires included mainly structured multiple choice questions and few very short answer questions. In addition, there were 4 validated questionnaires at the end of the survey. Mothers had the possibility of responding to these 4 questionnaires or to pass them. The questionnaire was first constructed through a web platform (www.sondageonline.com). Then, it was pretested by 6 women (2 of whom were mothers and 4 of whom were not mothers but who were used to developing questionnaires), which led to very minor changes.

#### Background variables

The first set of structured questions gathered information about various sociodemographic parameters that could influence the mothers’ experiences: age; socioeconomic status (combined level of education, professional status and household income (a subjective evaluation of the economic level was made by asking participants if they had the feeling that their financial resources allowed them to meet their household needs and was coded from very difficult to very easy; an objective evaluation was made by asking participants to provide their household’s monthly net income)); marital status; number of children; district of residence; ethnicity and Internet-related skills. Women were also asked if they worked in the medical or paramedical field and if they worked with children between 0 and 2 years of age.

Pregnancy and postnatal profiles were also investigated for the following items: history of last pregnancy (e.g., pregnancy monitoring, prenatal care, any health problem during the pregnancy) or childbirth and postnatal period (only for women who had already given birth: i.e., date of birth, gestational age, type of delivery, epidural, any problem during delivery, weight of the baby, history of the maternity stay); and type and experience of feeding. The experience of going back home after delivery was also investigated in women who had already given birth.

The couple relationship was also evaluated. Mothers were asked to describe the relationship with their partner (scale of 0–10: 0 = very negative, 10 = very positive) and to indicate how they would educate their baby (the possible answers were as follows: with the father of the baby, with my partner who is not the father of the baby, alone, with my wife (for a homosexual couple), or “other”, which required further).

#### Need for information

Mothers were first asked if they searched for information about pregnancy, about the maternity stay or, if they had already given birth, about postnatal issues (about the baby or themselves) and what information they searched for. Mothers were next asked whether they used the Internet to search for pregnancy or postnatal issues (about the baby or herself), whether they used other sources for searching information, what was their reasoning for this search strategy, whether they had one or more favorite websites to search, and the frequency with which they searched. They were also asked whether they found the information that they sought and if it was easy or difficult to find it. They were then requested to give a score on a 10-cm scale to assess how they felt about the reliability of the health information found on the Internet and to say if they had already visited a website verifying whether the information was incomplete or incorrect. The questionnaire also inquired how mothers used the information that they found (utility of the information) and whether their daily decision making was influenced by this information. Thus, the decision-making process was investigated. Indeed, the women were asked to rate their confidence in decision making (scale of 0–10) for their own health and for their child’s health both before and after using the Internet. In addition, the following question was asked to mothers: ‘Did the information that you found on the Internet influence the way you have thought of managing your child (e.g., diet, illness, sleep, games, pacifier, etc.)?” (scale of 0–10: 0 = not at all, 10 = absolutely).

Then, the reasons to search information on the Internet were also investigated. Following a review of the literature and previous studies conducted in our department, a list was created with different possible reasons for searching information from the Internet. These reasons were proposed to the respondents in the form of a structured questionnaire, and no space was given for additional explanations.

In addition, the questionnaire inquired whether the hospital in which they gave birth or were to give birth provided an online information tool to them and if they used it and were satisfied with it. The women were finally asked if they believed that health professionals should suggest suitable and reliable Internet websites where mothers could find relevant information about pregnancy or the postpartum period.

#### Need for psychological support

To explore the need for psychological support, mothers were asked, on a scale from 0 to 10, the following questions: if they felt supported by their companion, their family and their friends; if they felt alone, stressed, anxious and tired; and if they felt reassured in their (future) role of mothers. They were next asked if their research on the Internet has ever helped them to feel more supported or reassured in their role as (future) mothers and feel less alone, stressed, anxious or tired (the possible answers were as follows: not at all, a little, very, totally or “I do not feel concerned/I never did this kind of research on the Internet). Women were also asked if they experienced any stressful events during their pregnancy or since their delivery.

In addition, 3 validated questionnaires also evaluated the need for psychological support: the Postnatal Perceived Stress Inventory (PNPSI) for women who had already given birth [22] or the Antenatal Perceived Stress Inventory (APSI) for pregnant women [23], the Edinburg Postnatal Depression Scale (EPDS) [24] and the State-Trait Anxiety Inventory (STAI-Y1 SCALE) [25]. Finally, a fourth questionnaire (the Social Support Questionnaire; SSQ6) was also administered [26].

#### Need to share experiences

Mothers were then asked whether they felt the need to talk about their feelings around them and with whom they talked. They were asked, on a scale from 0 to 10, if they felt understood by the people they talked to about their feelings. The questionnaire also inquired whether mothers would like to talk more or less about their feelings and whether their research on the Internet had ever helped them talk further about it. Mothers were also asked whether they needed to talk with their friends, relatives or health professionals about the information they found on the Internet.

Mothers were next asked if they engaged in any hobbies or activities enabling them to take a break from parenting (the possible answers were as follows: not at all, not enough, enough, too much) and if their research on the Internet had already helped them to find these kinds of activities. If women had ever given birth, they were also asked if they went back to work. If they answered “yes”, the questionnaire inquired about their experiences, whether they were afraid prior to their return to work, and if their research on the Internet had helped them prepare for their return to work. If they answered “no”, the questionnaire only inquired if they were afraid to go back to work and if their research on the Internet had helped them to prepare for it.

#### Need for practical and material support

Mothers were then asked, on a scale from 0 to 10, if they felt supported in their housework and if they felt the need for help in completing their housework (the possible answers were as follows: much more, little more, enough, a little less, much less). If they said that they felt the need for help, a list of the more current housework was provided to the respondents, and they noted the tasks with which they needed help. The questionnaire also inquired whether anyone had already spontaneously proposed to help with housework, whether the women had already asked for help, and whether their research on the Internet had ever helped them in asking for help with housework.

### Promotion of the study and recruitment

Participants were recruited in various ways. In the first place, the study and the online link to questionnaires were widely published on various websites and dedicated Facebook pages. To reach as many women as possible, we chose to diversify the websites and Facebook pages by broadening out from official government ones to more individual ones, including Facebook pages from independent midwives and specific mother-and-baby shops. In addition, an internal email was sent twice to all members of the University of Liège – all specialties combined – to extend the dissemination of the study.

In the second place, in an effort to recruit also participants who did not have access to Facebook, flyers were placed in different stores for mothers and/or children. The study was also promoted by word of mouth – by mothers as well as by health professionals and early childhood professionals serving mothers of children aged 0 to 2 years – thereby allowing for a wider dissemination of the study. A list of the websites, dedicated Facebook pages and stores can be found in Table 1 with a free translation. The promotion of the study was made between August 7, 2017 and October 23, 2017 and was free.

**Table 1:** List of the websites, dedicated Facebook pages and stores used for the promotion of the study

**Original title**	**English free translation**
**Websites**	
Union Professionnelle des Sages-Femmes Belges (UPSFB)	Professional Union of Belgian Midwives
Wallonia e-Health Living Lab (WeLL)	Wallonia e-Health Living Lab (WeLL)
**Facebook pages**	
Baby-sitters de Belgique	Baby-sitters in Belgium
Bébékadom	= shop for mothers and babies
Le Ligueur	Le Ligueur
Les mamans qui déchirent	Mothers who Rock
Magicmaman	Magicmaman
Mamans et futures mamans de Belgique	Mothers and future mothers of Belgium
Parole de mamans	Mothers speak out
Plateforme pour une naissance respectée	Platform for a respected birth
Union Professionnelle des Sages-Femmes Belges (UPSFB)	Professional Union of Belgian Midwives
Wallonia e-Health Living Lab (WeLL)	Wallonia e-Health Living Lab (WeLL)
Santé Publique, Epidémiologie et Economie de la santé, Université de Liège	Public Health, Epidemiology and Health Economics, University of Liège
Club mamans	Moms club
Future maman pour 2017/2018 et déjà maman ou en essai bébé	Expectant mothers for 2017/2018 and already mom or mom trying to have a baby
ESP/ULB - Ecole de Santé Publique	Public Health school, University of Brussels
Fédération des centres pluralistes de planning familial	Federation of pluralistic family planning centers
Femmes et Santé	Womens and health
Wondermaman	Wondermothers
Ecotribu asbl	ASBL to support for proximal parenting
Naître à la Vie (Doula)	Being born to Life (Doula)
Liège... Ma ville...	Liège ... My city ...
Lune Après lune	Moon after moon
Au fil des lunes	As the moon go by
Accompagnement autour de la naissance	Psychological support in connection with birth
La Cigogne	The stork
Lunaissance	(Word play using moon and birth)
Ma Doula à Moi	My very own doula
En passant - doula	Along the way (doula)
Icosaèdre Mandarine	Mandarine Icosahedron
Les marraines de lait	The milk godmothers
Association Maman Blues	Association “Moms blues”
La profondeur des mères	The depth of mothers
**Stores**	
Z	Z
Orchestra	Orchestra
Bonbon Baby Store	Candy Baby Store
Dreambaby	Dreambaby

### Data collection

Once a woman responded to the questionnaire, she was enrolled in the study. Each time a woman replied to the survey, her answers appeared directly in the online database. The principal investigator had therefore to check her responses to verify the inclusion criteria. To ensure quality, questionnaires with less than 50 % of questions answered were excluded from the study.

### Data analysis

A Shapiro-Wilk test verified the normal distribution of all parameters and permitted the application of either parametric statistics or nonparametric statistics tests. All the data did not follow the normal distribution, but to provide a better understanding, all the quantitative variables are expressed as the mean ± standard deviation (SD), and qualitative variables are reported as relative (%) and absolute (n) frequencies. The evaluation of the participants’ demographic, pregnancy and postnatal profiles and the evaluation of each mother’s needs were realized by descriptive statistics. Chi-square and Mann–Whitney U tests facilitated the comparison of all these variables between pregnant women and women who had already given birth. The analyses were executed with the software *Statistica 13.3*. The results were considered statistically significant when the 2-tailed *p*-values were less than 0.05.

## Results

### Participants and sociodemographic characteristics

A total of 425 participants were enrolled in this study: 132 pregnant women and 293 women who had already given birth. Women were between 18 and 44 years old, and 48.7 % of them were primiparous. The participants’ demographic, pregnancy and postnatal profiles are presented in Table 2. There was no significant profile difference between pregnant women and women who had already given birth except for professional status and pregnancy care by an independent midwife.

**Table 2:** Participants’ demographic, pregnancy and postnatal profiles (N=425)

**Variable**	***n***	**Total**(*** n*** = 425)	**Pregnant women**(**n** = 132)	**Women who already gave birth **(***n*** = 293)	***p***-value
**Age of mothers (years; mean ± SD)**	425	31.1 ± 3.56	31.2 ± 3.54	31.0 ± 3.57	0.61
**Number of children (mean ± SD)**	425	1.69 ± 0.79	1.67 ± 0.81	1.70 ± 0.78	0.69
One child		*207 (48.7)*	*68 (51.5)*	*139 (47.4)*	
Two children		*153 (36.0)*	*42 (31.8)*	*111 (37.9)*	
Three children		*56 (13.2)*	*19 (14.4)*	*37 (12.6)*	
Four children		*8 (1.88)*	*3 (2.27)*	*5 (1.71)*	
Five children		*1 (0.24)*	*0 (0.00)*	*1 (0.34)*	
**Age of the last child (months; mean ± SD)**	293	-	-	3.36 ± 2.44	-
**Gestational age (weeks; mean ± SD)**	132	-	36.4 ± 2.68	-	-
**Pregnancy care**	425				
Private gynecologist		249 (58.6)	73 (55.3)	176 (60.1)	0.36
Gynecologist in a hospital		160 (37.6)	46 (34.8)	114 (38.9)	0.42
Independent midwife		135 (31.8)	56 (42.4)	79 (27.0)	0.002
Midwife attached to a hospital		40 (9.41)	9 (6.82)	31 (10.6)	0.21
General practitioner		21 (4.94)	4 (3.03)	17 (5.80)	0.20
Other		22 (5.18)	6 (4.55)	16 (5.46)	0.68
**Prenatal care **	424				0.56
Yes		258 (60.8)	83 (62.9)	175 (59.9)	
No		166 (39.2)	49 (37.1)	117 (40.1)	
**Health problems during pregnancy **	423				0.75
None		252 (59.6)	82 (62.1)	170 (58.4)	
One problem		115 (27.2)	33 (25.0)	82 (28.2)	
Two or more problems		56 (13.2)	17 (12.9)	39 (13.4)	
**Type of childbirth **	293	-	-		-
Vaginal delivery				242 (82.6)	
Programmed caesarean				28 (9.56)	
Emergency caesarean				23 (7.85)	
**Maternity length of stay (days; mean ± SD)**	292	-	-	3.75 ± 1.49	-
**Type of feeding (at the end of the maternity stay)**	292	-	-		-
Breastfeeding				231 (79.1)	
Bottle feeding				29 (9.93)	
Mixed feeding (breast + bottle)				32 (11.0)	
**Maternal health problems after delivery **	289	-	-		-
None				251 (86.9)	
One problem				34 (11.8)	
Two or more problems				4 (1.38)	
**Child health problems after delivery **	289	-	-		-
None				230 (79.6)	
One problem				41 (14.2)	
Two or more problems				18 (6.23)	
**Marital status (n (%))**	425				0.69
Married / In couple / In legal cohabitation (heterosexual)		413 (97.2)	129 (97.7)	284 (96.9)	
Married / In couple / In legal cohabitation (homosexual)		3 (0.71)	1 (0.76)	2 (0.68)	
Single / Divorced / Separated		6 (1.41)	2 (1.52)	4 (1.37)	
Widow		1 (0.24)	0 (0.00)	1(0.34)2 (0.68)	
Other		2 (0.47)	0 (0.00)		
**Living with her partner (n (%))**	425				0.62
Yes		417 (98.1)	130 (98.5)	287 (98.0)	
No		6 (1.41)	1 (0.76)	5 (1.71)	
No partner		2 (0.47)	1 (0.76)	1 (0.34)	
**Upbringing of children with (n (%))**	425				0.69
The father of the last children		415 (97.6)	129 (97.7)	286 (97.6)	
The wife		3 (0.71)	1 (0.76)	2 (0.68)	
Alone		5 (1.18)	2 (1.52)	3 (1.02)	
A companion who is not the father		2 (0.47)	0 (0.00)	2 (0.68)	
Other		0 (0.00)	0 (0.00)	0 (0.00)	
**Highest level of education completed (n (%))**	420				0.24
Primary and lower secondary		8 (1.90)	2 (1.53)	6 (2.08)	
Upper secondary		40 (9.52)	13 (9.92)	27 (9.34)	
Bachelor’s		177 (42.1)	48 (36.6)	129 (44.6)	
Nonuniversity long type		11 (2.62)	2 (1.53)	9 (3.11)	
Master’s		156 (37.1)	58 (44.3)	98 (33.9)	
PhD		26 (6.19)	8 (6.11)	18 (6.23)	
Other		2 (0.48)	0 (0.00)	2 (0.69)	
**Professional status (n (%)) **	421				< 0.0001
Active		151 (35.9)	68 (51.9)	83 (28.6)	
On leave (maternity or parental leave)		220 (52.3)	45 (34.6)	175 (60.3)	
In training		7 (1.66)	3 (2.29)	4 (1.38)	
Unable to work (disabled or illness)		13 (3.09)	7 (5.34)	6 (2.07)	
Housewife		9 (2.14)	1 (0.76)	8 (2.76)	
Unemployed		19 (4.51)	7 (5.34)	12 (4.14)	
Other		2 (0.48)	0 (0.00)	2 (0.69)	
**(Para)medical profession (n (%)) **	419				0.80
Yes		135 (32.2)	43 (33.1)	92 (31.8)	
No		284 (67.8)	87 (66.9)	197 (68.2)	
**Profession in contact with children 0-2 years of age (n (%)) **	421				0.89
Yes		82 (19.5)	25 (19.1)	57 (19.7)	
No		339 (80.5)	106 (80.9)	233 (80.3)	
**Subjective economic level (n (%)) **	420				0.07
Very easy to meet household needs		53 (12.6)	16 (12.2)	37 (12.8)	
Easy to meet household needs		226 (53.8)	82 (62.6)	144 (49.8)	
Difficult to meet household needs		111 (26.2)	25 (19.1)	86 (29.8)	
Very difficult to meet household needs		30 (7.14)	8 (6.11)	22 (7.61)	
**Household income per month (n (%)) **	421				0.57
Less than €1000		2 (0.48)	1 (0.76)	1 (0.34)	
Between €1000 and €1999		47 (11.2)	14 (10.7)	33 (11.4)	
Between €2000 and €2999		78 (18.5)	28 (21.4)	50 (17.2)	
Between €3000 and €3999		184 (43.7)	58 (44.3)	126 (43.4)	
€4000 and more		78 (18.5)	23 (17.6)	55 (19.0)	
Does not know		4 (0.95)	0 (0.00)	4 (1.38)	
Refused to answer		28 (6.65)	7 (5.34)	21 (7.24)	

### Exploration of needs

#### Need for information

The need for information was widely present in the population (Table 3): 95.7 % of participants reported needing to search for information at least once during pregnancy and/or after delivery. The majority of the pregnant women (92.4 %) and the new mothers (90.1 %) admitted seeking information about the pregnancy. After childbirth, many new mothers (84.6 %) also reported seeking information about themselves or about their baby (65.4 % both for themselves and the baby, 16.4 % for the baby only, and 2.74 % for themselves only). However, this need seemed to be significantly greater in pregnant women (92.4 %) than in mothers (84.6 %, *p *= 0.03). The majority of women who seek information (91.4 %) reported that they used the Internet to perform their research, whether it was during or after the pregnancy (*p *= 0.18). Nevertheless, women seem to search for information significantly (*p *< 0.0001) more frequently on the Internet after giving birth (54.8 % reported “very often” or “daily”) than during pregnancy (41.2 % reported “very often” or “daily”). Women judged the search for information on the Internet in a similar manner (i.e., ease of accessing information, quality of the information, utility of information), whether they were pregnant or had already given birth (*p* > 0.05). Finally, pregnant women (77,1 %) and mothers (73,8 %) were similarly interested in the recommendation of reliable websites (*p* = 0.47).

**Table 3:** Exploration of the need for information among pregnant women and mothers

**Variables**	**n**	**Total population**	**Women who had already given birth**	**Pregnant women**	***p***
**Seeking perinatal information at least once during pregnancy and/or after delivery (n (%))**	423		-*	-*	-*
Yes		405 (95.7)			
No		18 (4.26)			
**Seeking perinatal information during pregnancy OR after delivery (n (%))**	423				0.03
Yes		368 (87.0)	247 (84.6)	121 (92.4)	
No		55 (13.0)	45 (15.4)	10 (7.63)	
**Using the Internet to seek for information (n (%))**	407				0.18
Yes		372 (91.4)	257 (90.2)	115 (94.3)	
No		35 (8.60)	28 (9.82)	7 (5.74)	
**Frequency of the use of the Internet (n (%))**	371				<0.0001
Each day		35 (9.43)	26 (10.1)	9 (7.89)	
Very often		153 (41.2)	115 (44.7)	38 (33.3)	
Often		97 (26.1)	53 (20.6)	44 (38.6)	
Sometimes		86 (23.2)	63 (24.5)	23 (20.2)	
**Easy or difficult to find information on the Internet (mean ± SD/10; very difficult=0 – very easy=10)**	371	6.74 ± 1.83	6.65 ± 1.85	6.94 ± 1.77	0.12
**Finding incomplete information on the Internet (n (%))**	371				0.12
Always		21 (5.66)	17 (6.61)	4 (3.51)	
Often		245 (66.0)	175 (68.1)	70 (61.4)	
Rarely		100 (27.0)	63 (24.5)	37 (32.5)	
Never		5 (1.35)	2 (0.78)	3 (2.63)	
**Finding false information on the Internet (n (%))**	371				0.49
Always		6 (1.62)	5 (1.95)	1 (0.88)	
Often		135 (36.4)	95 (37.0)	40 (35.1)	
Rarely		215 (56.0)	149 (58.0)	66 (57.9)	
Never		15 (4.04)	8 (3.11)	7 (6.14)	
**Quality of information on the Internet (mean ± SD/10; very bad=0 – very good=10)**	371	5.29 ± 1.48	5.30 ± 1.42	5.26 ± 1.61	0.99
**Utility of information on the Internet (mean ± SD/10; very useless=0 – very useful=10)**	369	6.08 ± 1.59	6.07 ± 1.61	6.12 ± 1.54	0.69
**Influence of the information found on the Internet on the management of the child (mean ± SD/10; very poor=0 – very strong=10)**	253	4.43 ± 2.37	4.43 ± 2.37	-	-
**Reasons for searching for information on the Internet (n/N (%))**	/				
- To determine information “on their own”		316/362 (87.3)	220/252 (87.3)	96/110 (87.3)	0.99
- To check for information about specific maternal or child symptoms		268/364 (73.6)	177/253 (70.0)	91/111 (82.0)	0.02
- To have more control over decisions affecting their own health or their child’s health		156/360 (43.3)	115/252 (45.6)	41/108 (38.0)	0.18
- Because a health care provider had recommended a particular website		31/355 (8.73)	21/248 (8.47)	10/107 (9.35)	0.30
- Because they found one or more well-designed websites that made them want to read information		216/358 (60.3)	152/250 (60.8)	64/108 (59.3)	0.31
- To acquire information in addition to that already provided by health professionals		253/362 (69.9)	184/252 (73.0)	69/110 (62.7)	0.05
- Because the information provided by a health professional was not clear		79/358 (22.1)	62/251 (24.7)	17/107 (15.9)	0.07
- Because the information provided by a health professional was not satisfactory		72/356 (20.2)	59/250 (23.6)	13/106 (12.3)	0.01
- Because of a lack of time to ask a health professional questions		118/358 (33.0)	88/249 (35.3)	30/109 (27.5)	0.15
- Because women did not feel comfortable or did not dare to ask questions to their healthcare professional		93/357 (26.1)	74/249 (29.7)	19/108 (17.6)	0.02
- To gain confidence to speak to a health professional about a concern		61/355 (17.2)	45/248 (18.1)	16/107 (15.0)	0.25
- To have a second opinion		190/356 (53.4)	141/249 (56.6)	49/107 (45.8)	0.06
**Thought that professionals should suggest suitable websites**	421				0.47
Yes		315 (74.8)	214 (73.8)	101 (77.1)	
No		106 (25.2)	76 (26.2)	30 (22.9)	

*Not tested.

#### Need for psychological support

Women generally seemed to be quite satisfied with the support they received (Table 4). They attributed an average score of 7.70 ± 2.17/10 to the support provided by their partner, 7.42 ± 2.31/10 to the support provided by their family and 7.28 ± 2.19/10 to the support provided by their friends. However, this feeling weakened during the postpartum period. On the one hand, after giving birth, women gave lower ratings to the support they received from their family (*p* = 0.05) and their friends (*p* = 0.01) than did pregnant women. In addition, they assigned an average score of 7.12 ± 2.17/10 when they had to rate the relationship they had with their partner, while pregnant women rated it with a mean score of 7.94 ± 1.73/10 (*p* < 0.0001). Moreover, the social support scores regarding availability (20.6 ± 10.5/54 vs. 23.7 ± 11.5/54) and satisfaction (28.5 ± 6.16/36 vs. 30.1 ± 5.38/36) were significantly higher during pregnancy than during the postpartum period (*p* = 0.01).

**Table 4:** Exploration of the need for psychological support among pregnant women and mothers

**Variables**	***n***	**Total population**	**Women who had already given birth**	**Pregnant women**	***p***-value
**Physical state (mean ± SD/10; very bad=0 - very well=10)**	423	6.57 ± 2.24	6.59 ± 2.25	6.53 ± 2.24	0.77
**Psychological state (mean ± SD/10; very bad=0 - very well=10)**	423	6.92 ± 2.15	6.79 ± 2.19	7.20 ± 2.04	0.09
**Feeling supported by companion (mean ± SD/10; not at all=0 - totally=10)**	419	7.70 ± 2.17	7.65 ± 2.24	7.80 ± 2.02	0.74
**Definition of the relationship with the companion (mean ± SD/10; very negative=0 - very positive=10)**	419	7.37 ± 2.08	7.12 ± 2.17	7.94 ± 1.73	<0.001
**Feeling supported by family (mean ± SD/10; not at all=0 - totally=10)**	423	7.42 ± 2.31	7.28 ± 2.34	7.72 ± 2.23	0.05
**Feeling supported by friends (mean ± SD/10; not at all=0 - totally=10)**	422	7.28 ± 2.19	7.06 ± 2.31	7.78 ± 1.80	0.01
**Feeling alone (mean ± SD/10; not at all=0 - totally=10)**	423	3.01 ± 2.65	3.35 ± 2.67	2.25 ± 2.46	<0.0001
**Feeling stressed (mean ± SD/10; not at all=0 - totally=10)**	423	3.95 ± 2.64	4.01 ± 2.66	3.81 ± 2.60	0.56
**Feeling anxious (mean ± SD/10; not at all=0 - totally=10)**	423	3.51 ± 2.72	3.50 ± 2.76	3.53 ± 2.62	0.68
**Feeling tired (mean ± SD/10; not at all=0 - totally=10)**	423	3.62 ± 2.34	3.47 ± 2.22	3.95 ± 2.57	0.11
**Feeling reassured in her role as a mother (mean ± SD/10; not at all=0 - totally=10)**	423	7.22 ± 2.11	7.30 ± 2.07	7.04 ± 2.21	0.28
**Depression scale (EPDS: mean ± SD/30)**	369	9.15 ± 5.54	9.63 ± 5.53	8.19 ± 5.45	0.01
**Stress scale**					
PNPSI scale (mean ± SD/95)	248	41.9 ± 12.1	41.9 ± 12.1	-	-
APSI scale (mean ± SD/60)	123	26.3 ± 6.08	-	26.3 ± 6.08	
**Anxiety scale (STAI-Y1 scale: mean ± SD/80)**	366	39.7 ± 12.3	40.8 ± 12.9	37.7 ± 10.9	0.07
**Social support (SSQ6)**					
Availability score (mean ± SD/54)	362	21.6 ± 10.9	20.6 ± 10.5	23.7 ± 11.5	0.01
Satisfaction score (mean ± SD; score from 6 to 36)	360	29.0 ± 5.95	28.5 ± 6.16	30.1 ± 5.38	0.01

Additionally, feelings of loneliness were higher during the postpartum period than during pregnancy (3.35 ± 2.67/10 vs. 2.25 ± 2.46/10; *p* < 0.0001). Furthermore, depression scores (9.63 ± 5.53/30 vs. 8.19 ± 5.45/30; *p* = 0.01) were significantly higher during the postpartum period than during pregnancy.

#### Need to share experiences

The majority of women (97.4 %) said that they talk about their feelings with their relatives or with professionals (53.4 % “sometimes”, 33.3 % “often” and 10.9 % “very often”). The need to share experiences (Table 5) thus seemed to be present among both pregnant women and mothers (*p* = 0.50). Sources of sharing experiences are different between groups: not surprisingly, pregnant women discussed more with midwives and gynecologists than did mothers (*p *< 0.0001). When women shared their feelings, they tended to think that people around them understand them (7.76 ± 1.74/10) whether it was during pregnancy or after childbirth (*p* = 0.10). However, many women would have liked to have the opportunity to talk more about their feelings, and this need seems to be more pronounced in the postpartum period (47.6 %) than during pregnancy (30.6 %, *p* = 0.01). Compared to pregnant women, recent mothers (84.6 % vs. 62.6 %, *p* < 0.0001) believed that they did not have enough leisure activities.

**Table 5:** Exploration of the need to share experiences among pregnant women and mothers

**Variables**	**n**	**Total population**	**Women who had already given birth**	**Pregnant women**	***p***-value
**Discussion about what she feels or experiences (n (%))**	423				0.50
Never		10 (2.36)	8 (2.74)	2 (1.53)	
Sometimes		226 (53.4)	160 (54.8)	66 (50.4)	
Often		141 (33.3)	96 (32.9)	45 (34.4)	
Very often		46 (10.9)	28 (9.59)	18 (13.7)	
**With whom? (n (%))**	413				
Companion		382 (92.5)	260 (91.5)	122 (94.6)	0.28
Midwife		137 (33.2)	73 (25.7)	64 (49.6)	<0.0001
Gynecologist		91 (22.0)	33 (11.6)	58 (45.0)	<0.0001
GP		32 (7.75)	24 (8.45)	8 (6.20)	0.43
Pharmacist		11 (2.66)	8 (2.82)	3 (2.33)	0.77
Family		279 (67.6)	187 (65.8)	92 (71.3)	0.27
Friends with children		346 (83.8)	239 (84.2)	107 (82.9)	0.78
Friends without children		155 (37.5)	98 (34.5)	57 (44.2)	0.06
Other mothers she does not necessarily know well		50 (12.1)	33 (11.6)	17 (13.2)	0.65
Other mothers she has never met *(ex: forums, Facebook groups)*		70 (16.9)	54 (19.0)	16 (12.4)	0.10
Others		14 (3.39)	6 (2.11)	8 (6.20)	0.02
**Feeling understood by these persons (mean ± SD/10; not at all=0 - totally=10)**	406	6.76 ± 1.74	6.65 ± 1.79	7.02 ± 1.60	0.10
**Want to speak more about her feelings (n (%))**	423				0.01
Much more		34 (8.04)	30 (10.3)	4 (3.05)	
A little more		145 (34.3)	109 (37.3)	36 (27.5)	
Speak enough		236 (55.8)	149 (51.0)	87 (66.4)	
A little less		6 (1.42)	3 (1.03)	3 (2.29)	
Much less		2 (0.47)	1 (0.34)	1 (0.76)	
**Discussion about the information found on the Internet (n (%))**	423				0.01
Never		13 (3.07)	6 (2.05)	7 (5.34)	
Sometimes		222 (52.5)	148 (50.7)	74 (56.5)	
Often		147 (34.8)	112 (38.4)	35 (26.7)	
Always		23 (5.44)	18 (6.16)	5 (3.82)	
No research on the Internet		18 (2.46)	8 (2.74)	10 (7.63)	
**With whom? (n (%))**	395				
Companion		365 (92.4)	258 (91.8)	107 (93.9)	0.49
Family		265 (67.1)	188 (66.9)	77 (67.5)	0.90
Friends		265 (67.1)	182 (64.8)	83 (72.8)	0.12
Midwife		117 (29.6)	73 (26.0)	44 (38.6)	0.01
ONE		53 (13.4)	52 (18.5)	1 (0.88)	<0.0001
Pediatrician		77 (19.5)	74 (26.3)	3 (2.63)	<0.0001
Gynecologist		80 (20.3)	46 (16.4)	34 (29.8)	0.003
GP		33 (8.35)	29 (10.3)	4 (3.51)	0.03
Pharmacist		43 (10.9)	35 (12.5)	8 (7.02)	0.12
Others		6 (1.52)	2 (0.71)	4 (3.51)	0.04
**Hobbies / leisure activities (n (%))**	423				<0.0001
Not at all		91 (21.5)	77 (26.4)	14 (10.7)	
Not enough		238 (56.3)	170 (58.2)	68 (51.9)	
Enough		91 (21.5)	45 (15.4)	46 (35.1)	
Too much		3 (0.71)	0 (0.00)	3 (2.29)	

#### Need for practical and material support

Women gave a mean score of 6.04 ± 2.69/10 in feeling supported in their household chores, whether during or after pregnancy (*p *= 0.11). The need for practical support (Table 6) therefore seems to be present around the time of birth, and it seems to be even more pronounced during the postpartum period than during pregnancy. Indeed, women who gave birth (72.5 %) would like to have more help with housework chores than pregnant women (80.9 %; *p*= 0.01) and for more types of housework chores (4.45 ± 2.54 vs. 3.92 ± 2.79; *p*= 0.047). Women often did not dare to seek the help they needed, and it seemed that the mothers’ relatives offered their help significantly more often to pregnant women (81.7 %) than to new mothers (71.8 %, *p* = 0.02).

**Table 6:** Exploration of the need for practical and material support among pregnant women and mothers

**Variables**	***n***	**Total population**	**Women who had already given birth**	**Pregnant women**	***p***-value
**Feeling supported in housework (mean ± SD/10; not at all=0 - totally=10)**	423	6.04 ± 2.69	5.89 ± 2.75	6.39 ± 2.54	0.11
**Need help with housework (n (%))**	423				0.01
Much more		78 (18.4)	65 (22.3)	13 (9.92)	
A little more		253 (59.8)	171 (58.6)	82 (62.6)	
Enough		91 (21.5)	55 (18.8)	36 (27.5)	
A little less		1 (0.24)	1 (0.34)	0 (0.00)	
Much less		0 (0.00)	0 (0.00)	0 (0.00)	
**Amount of housework (number of chores: mean ± SD/10)**	401	4.28 ± 2.63	4.45 ± 2.54	3.92 ± 2.79	0.047
**Already spontaneously proposed help for housework? (n (%))**	422				0.02
Very often		33 (7.82)	17 (5.84)	16 (12.2)	
Often		67 (15.9)	39 (13.4)	28 (21.4)	
Sometimes		120 (28.4)	86 (29.6)	34 (26.0)	
Very rarely		96 (22.7)	67 (23.0)	29 (22.1)	
Never		106 (25.1)	82 (28.2)	24 (18.3)	
**Already asked for some help for housework? (n (%))**	421				0.06
Always		12 (2.85)	10 (3.44)	2 (1.54)	
Often		46 (10.9)	29 (9.98)	17 (13.1)	
Sometimes		215 (51.1)	139 (47.8)	76 (58.5)	
Never		148 (35.2)	113 (38.8)	35 (26.9)	

## Discussion

The aim of this study was to explore maternal needs by comparing the needs of mothers with those of pregnant women.

The need for information is largely present around the time of birth, and this need has already been proven in several studies [27–30]. Some studies showed that new mothers (having their first baby) felt unprepared for motherhood [4, 28, 31]. They fluently searched for reliable and realistic information [27, 30] and appreciated having testimonials from other mothers with which they could compare their experiences [10] and appease their fears and anxieties [17]. The need for information seemed to be more present during pregnancy than during the postpartum period. Nevertheless, women used the Internet significantly more often to search for information after childbirth than during pregnancy. Regarding the results of our previous study [10], this finding may not be so surprising. Indeed, although the need for information is more important during pregnancy, it is still important after childbirth. However, professionals are aware of the important need for information during pregnancy, but they believe that this need disappears after delivery [10]. It is therefore not surprising that the efforts of professionals are more concentrated during pregnancy. Thus, mothers may feel left out after giving birth and turn to the Internet to bridge gaps. This hypothesis is supported by the difference in information sources, showing that pregnant women discussed significantly more information with midwives and gynecologists than did mothers.

The need for psychological support seemed to be more important after childbirth than before childbirth. Indeed, even if the global satisfaction with psychological support was fairly good, it weakened after childbirth. Feelings of loneliness and depression scores were also higher during the postpartum period than during pregnancy. Some studies have already highlighted the social upheavals that the arrival of a child generates [3, 4]. In addition, the process of motherhood is described by some authors as a period of identity crisis that can be compared to adolescence by the movement it imposes on the women’s personality [32, 33]. It is therefore not surprising that women need to be surrounded and supported by people they consider to be important around them [18, 19].

Thus, the needs for psychological support and to share experiences seemed to be closely linked. Sharing experiences seem to be a form of psychological support. Indeed, mothers like to have the possibility of discussing what they experience, especially with other mothers, to determine if what they are experiencing is normal [10]. The concept of normality seems very important in this period of life. Sharing experiences helps mothers to address this fear of abnormality. This step seems to be very important in the appropriation of the role of a mother. Women need empathy and to be understood. Moreover, this study showed that more women would like to have the opportunity to talk more about their feelings during the postpartum period than during the pregnancy. Once again, this study highlighted the importance of social support around a birth [20, 34–36].

The need for practical support is also present around a birth, and it seems to be more pronounced during the postpartum period than during pregnancy. Indeed, housework chores are indispensable, but it seems to be a considerable challenge for mothers [10, 20, 37]. Already tired and worried about the management of their child, mothers would like more help in household management, at least in the first weeks after childbirth, to have the time to adapt themselves to their new situation [20, 38].

### Implications for practice

There is a discrepancy between the professionals’ perception of maternal needs and the needs felt by mothers [10]. Indeed, professionals seem to be more concerned about the needs during pregnancy than during the postpartum period. However, this study and many other studies showed that mothers present important physical and emotional needs during the postpartum period [3, 4, 10, 17–20, 27–31, 33, 37–44]. Thus, the promotion of maternal health by professionals cannot end at the birth of the newborn [38]. For a healthy postnatal period, women must use their own skills to ensure that their needs and those of their family are met [38]. Postnatal care providers therefore must help women find necessary resources to develop the required skills to appropriately meet their needs. They must respect a holistic vision of maternal health promotion, especially in the context of shortened maternity leave [45, 46]. For example, as a part of our research project, we designed a new website (www.supermaman.be). This website is intended to meet the needs of mothers (but also parents) by distributing and centralizing quality information from the last trimester of pregnancy until 1 year after childbirth. The addressed themes within the website were agreed by a Delphi method bringing together parents as well as health and early childhood professionals [47]. The content of this website was then produced in collaboration with professionals and experts on the topics covered.

### Strengths and limitations

To our knowledge, this is the first study to explore maternal needs during the postpartum period by comparing needs during and after pregnancy. Additionally, this study is a continuation of a qualitative investigation of the mothers’ needs. The qualitative method was well adapted to explore the mothers’ needs and to obtain very rich results. However, qualitative methods did not allow for the measurement of the frequency or the extent to which mothers felt these needs. A quantitative investigation was therefore necessary to complete the mothers’ needs exploration.

This study also presented some possible limitations. This survey used a self-report questionnaire that induced all the estimation errors that such a questionnaire can cause [48]. This study used a web survey, and we could not record the number of nonparticipants to the survey. Moreover, the present study only investigated the needs of mothers who gave birth to healthy term babies. The representativeness of the respondents therefore cannot be asserted for all mothers in Belgium. Nevertheless, the survey was available on many websites and social networks to reach a large number of Belgian mothers (n = 425), and the results seemed to correspond with those of other studies conducted during pregnancy. Indeed, a recent systematic review sowed that there is growing evidence to suggest that Facebook is a useful recruitment tool and that its use should be considered when implementing health research [49]. It seems to reduce costs and recruitment periods but also to provide a better representation of the population in comparison with traditional recruitment methods (print, radio, television, and email).

## Conclusions

The postnatal period can be described as a period of identity crisis in which mothers must address new needs. This study allowed us to confirm, on a large scale, that the 4 categories of needs identifying earlier in our previous study [10] (needs for information, for psychological support, for the sharing of experiences and for practical and material support) are inherent to the postpartum period. Indeed, all these needs are also experienced during pregnancy but in a lesser intensity or in a less specific way. For example, although pregnant women seem to feel a greater need for information, recent mothers turn more to the internet, which would reflect a greater lack of information after childbirth. The other 3 needs are more present in the postpartum period: the feeling of not being enough socially supported seems higher and the need of some opportunity to talk about their feelings as well as the need for practical support seem to be more pronounced. This study helps to clarify what mothers experience after childbirth. Trying to meet these needs could offer an opportunity to improve the quality of life of mothers and to prevent the risk of psychological distress that may occur after childbirth.

## Data Availability

The dataset on which the conclusions of this manuscript rely will not be made available given the conditions stated in the written informed consent form to protect the identity of the participants.

## Abbreviations

APSI: Antenatal Perceived Stress Inventory
EPDS: Edinburg Postnatal Depression Scale
PNPSI: Postnatal Perceived Stress Inventory
SD: Standard Deviation
SSQ6: Social Support Questionnaire
STAI-Y1 SCALE: State-Trait Anxiety Inventory
